# Towards on-the-fly data post-processing for real-time tomographic imaging at TOMCAT

**DOI:** 10.1186/s40679-016-0035-9

**Published:** 2017-01-03

**Authors:** Federica Marone, Alain Studer, Heiner Billich, Leonardo Sala, Marco Stampanoni

**Affiliations:** 1 Swiss Light Source, Paul Scherrer Institute, Villigen, Switzerland; 2 Information Technology Division, AIT, Paul Scherrer Institute, Villigen, Switzerland; 3 Institute for Biomedical Engineering, University and ETH Zurich, Zurich, Switzerland

**Keywords:** High data rates, Fast tomographic reconstruction, Ultrafast X-ray tomographic imaging, Tomographic microscopy beamline, Efficient pipeline

## Abstract

Sub-second full-field tomographic microscopy at third-generation synchrotron sources is a reality, opening up new possibilities for the study of dynamic systems in different fields. Sustained elevated data rates of multiple GB/s in tomographic experiments will become even more common at diffraction-limited storage rings, coming in operation soon. The computational tools necessary for the post-processing of raw tomographic projections have generally not experienced the same efficiency increase as the experimental facilities, hindering optimal exploitation of this new potential. We present here a fast, flexible, and user-friendly post-processing pipeline overcoming this efficiency mismatch and delivering reconstructed tomographic datasets just few seconds after the data have been acquired, enabling fast parameter and image quality evaluation as well as efficient post-processing of TBs of tomographic data. With this new tool, also able to accept a stream of data directly from a detector, few selected tomographic slices are available in less than half a second, providing advanced previewing capabilities paving the way to new concepts for on-the-fly control of dynamic experiments.

## Background

Sub-second tomographic experiments at third-generation synchrotron sources are becoming reality, thanks also to recent developments of detection systems combining CMOS technology with sustained high data rate streaming [1]. The visualization and investigation of dynamic processes in 3D through time is now possible, opening new possibilities in different disciplines ranging from materials (e.g., [2]) to biological sciences (e.g., [3, 4]). Time-resolved 3D snapshots of dynamic systems are important for the validation of theoretical models until recently often extrapolated from 2D information. Tomographic experiments with sub-second time resolution can also provide a look at phenomena in 3D, never observed so far due to lack of adequate methods.

To fully exploit these recent technological achievements, the IT infrastructure needs to be matched to these high and sustained data rates. In addition to specific solutions for efficiently streaming data at elevated rates and storing large amounts of data, requirements are also high for the post-processing part. Optimal control of fast tomographic experiments at synchrotrons requires fast access to reconstructed tomographic datasets. Both beamline and experimental parameters can in this way be adjusted and fine-tuned in a timely manner so that they maximize image quality. The time scales, dynamic properties, and sequences for many phenomena never investigated in 3D so far are often not known before the experiment. Pre-characterization of these systems through previewing capabilities is needed for establishing adequate acquisition protocols. Although 2D projections of an evolving system can provide insightful information, this tool might not be sufficient if complex structures are present or high density sensitivity is required. Rapid availability of a selection of reconstructed tomographic slices during the experiment can strongly facilitate its control also through on-the-fly adjustments of the relevant parameters (e.g., temperature).

At the TOMCAT beamline [5] at the Swiss Light Source, during the past few years a dedicated end station for ultrafast tomographic microscopy has been established [6] featuring the unique detector system GigaFRoST [1]. This system can be read out continuously in an unlimited manner leading to sustained data rates as high as 7.7 GB/s. To fully exploit the potential provided by this innovative system, a new and efficient tomographic reconstruction pipeline has been developed. Although several solutions at other facilities exist (e.g., TomoPy at APS [7], Savu at DLS [8], SPOT at ALS [9], PyHST at the ESRF [10], UFO at KIT [11, 12]), peculiarities of the local IT infrastructures as well as specific goals led to the development and implementation of a new pipeline. The design of this new framework aims primarily at computational efficiency for fast reconstruction at the beamline during experiments taking advantage of a dedicated cluster. It however also needs to provide flexibility and easy access to the code for non IT-experts such as beamline scientists to ensure possibilities for growth of the offered capabilities with time. The computational hardware landscape at the Swiss Light Source is dominated by CPU power. A GPU solution is not considered favorable in particular because of need for specialized know-how for software development and implementation, currently not available in-house. The developed and presented framework does however not preclude the future use of GPUs.

In the following sections, we discuss the data format chosen before describing the different aspects of the developed post-processing pipeline. We conclude with a detailed performance assessment.

## Methods

### Data format

Access to rather small files as well as reading and writing small chunks (few kB) of data is, in general, largely inefficient and should be avoided to fully exploit the potential of modern shared file systems. This was exactly the case, when each single tomographic projection was stored as a separate (TIFF) file, as until recently typically done at most tomographic microscopy beamlines around the world, to directly take advantage of APIs for commercial detectors. For high efficiency, few large files (6–8 GB) are instead recommended, where data are read or written in large chunks (MB).

In this context, an optimized data format has been selected permitting fast I/O and compatibility with data from other synchrotron sources: we adopted the scientific data exchange format [13], based on the HDF5 technology [14]. This technology, a versatile data model for very complex data objects and metadata, is particularly suited to push I/O efficiency. There are no limitations on file size and on the number of objects stored in a file. It integrates features to maximize access time performance and storage space optimization.

In our current implementation, the raw data are written to an HDF5 file on disk in a sequential way using the direct chunk write function [15] and an n-bit filter. The HDF5 technology also supports parallel writing. We have so far not exploited this feature, to keep maximum flexibility with regard to possible compression approaches, currently under investigation for tomographic data. It could however be integrated in the current framework, if increased writing performance will be required.

The reconstruction pipeline reads instead the raw data from file in a parallel fashion. The theoretical limit of 5 GB/s (related to our current gpfs file server) has been demonstrated while reading from a large HDF5 file using the Python *h5py* library [16]. The used chunking strategy is optimized for fast single frame access, the most natural and general approach for tomographic data. Other options, for specific applications (e.g., absorption tomography), could be advantages and are under evaluation.

### Pipeline description

#### Main core

A typical full-field tomographic dataset acquired in few minutes at a third-generation synchrotron source consists of few thousand angular views (e.g., 1500–2000), each with more than 2000 × 2000 pixels, and a collection of dark- and white- (or flat-) field images used for normalization. Such raw dataset routinely exceeds 16 GB.

**Fig. 1:**
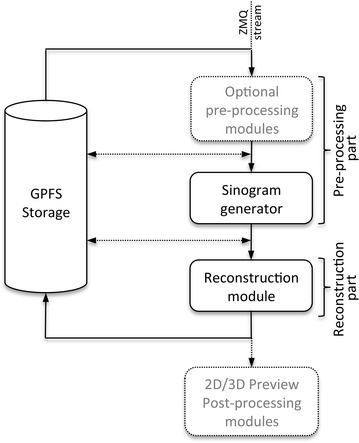
Diagram illustrating the main blocks and flow of the post-processing pipeline (*solid lines*). *Dash lines* indicate optional modules (e.g., phase retrieval) and actions (e.g., writing sinograms to file)

The post-processing pipeline consists of 2 main blocks: a pre-processing part generating the sinograms and the tomographic reconstruction function itself (Fig. 1).

##### Sinogram generator

In this first step, each angular view is corrected for the dark current of the detector and the background is normalized using the average of the acquired white-field images. In addition, the dataset is reorganized into sinograms, each containing the necessary information to reconstruct a selected tomographic slice. If this operation is performed in a naïve way, all projection images need to be open and a small chunk of data needs to be read to generate a single sinogram resulting in poor scalability due to the high I/O load. Furthermore if the generation of the sinograms for a typical dataset (usually in the order of 2000) is completely parallelized, this step would result in 1500 × 2000 simultaneous random accesses to the shared file system where the angular projections are stored, definitely a non-optimized procedure quickly resulting in a bottleneck, in particular for the high data rates of cutting-edge detectors. To overcome this bottleneck, here MPI has been used. Larger chunks of raw data are read and sent to the dedicated computing nodes at once, significantly improving the performance. The read/compute core ratio is determined empirically. A ratio between 1:6 and 1:8 is advantageous for medium size clusters. For larger clusters, this ratio will be smaller (it is not optimal to have many reading cores, reading just little data), for smaller systems it will be larger, to avoid having just a single reading core. It is important in particular for memory reasons that the reader cores are spread evenly across the nodes within the cluster (equal number on each node).

**Fig. 2:**
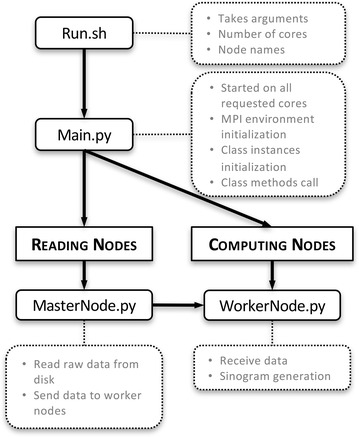
Skeleton of the sinogram generator package with the main software modules and their main tasks

Figure 2 shows the skeleton of the developed sinogram generation software. The main application is started on all requested cores and performs MPI environment and class instance initializations. Based on the MPI process rank of each core, it is decided if it is a reading or computing core and the corresponding class method is called. The assigned reading cores read then the raw data from disk. These data are sent to the computing cores, which generate the sinograms.

The computed sinograms can either be written to disk or piped directly into the tomographic reconstruction software. In this latter case, at least the correct center of rotation needs to be known to ensure high quality tomographic reconstructions. Therefore an additional routine, to be run prior to the sinogram generation, has also been developed. This routine runs just on one single node, using though all available cores. It computes, following [17], an estimation of the center of rotation and any dependency of this number on the sinogram within a dataset. If the center of rotation varies as a function of the sinogram number, implying an imperfect experimental alignment, the projections can be rotated according to the computed angle to compensate for the misalignment. For tomographic scans performed with the rotation axis positioned at the side of the available field of view, with the aim of doubling the size of the sample, which can be accommodated in an experiment without the need to resort to local tomography, the mentioned routine also provides the projection overlap. This is an important figure for the automatic stitching of projections acquired at angular positions spaced by 180°. All these estimated parameters are written together with relevant scan information (e.g., number of projections) to a log file, where they are accessible to the sinogram generator run in the next step in the pipeline.

##### Tomographic reconstruction algorithm

Although in the future we plan to expand the reconstruction capabilities including selected iterative algorithms, the post-processing pipeline as currently implemented at TOMCAT exclusively uses *gridrec* [18]. Despite being based on the Fourier Transform method, this fast analytic tomographic reconstruction algorithm has been validated as a valuable alternative to standard filtered back projection routines. The advantage of Fourier techniques lies in their intrinsic smaller number of required operations compared to other analytical methods. *Gridrec* is highly optimized for conventional CPU technology, not requiring more specialized architectures such as GPUs, to achieve a competitive reconstruction speed.

For integration in the pipeline, the original code has been adjusted to be compatible with multi-processing. For maximum flexibility two instances of the same function have been created. To permit the tomographic reconstruction of existing sinograms stored on the file system, the *gridRecMPIWrapper* launches as many instances of a *gridrec* standard executable as needed to process all sinogram files. To instead reduce the I/O load and for highest speed, the gridrec C code compiled as shared library is loaded from Python, so that the sinograms can be delivered to the reconstruction routine directly from memory.

The pipeline framework has been conceived in a modular way enabling the integration of additional pre- and post-processing steps at a later stage, as they might appear in the literature, in an easy manner. Currently available is a routine suppressing anomalously bright spots (zingers) typically observed on projection data when intense polychromatic radiation is used. They are the consequence of scattered X-ray photons hitting the detector chip directly and depositing significantly more energy than visible light photons. Zingers translate into tomographic reconstructed slices as lines. The removal routine, inspired by [19], works on sinograms, isolates the anomalous pixels by thresholding and substitutes them through an interpolation scheme. Two functions addressing ring artifacts are also included, more will be offered in the future. Concentric (half) rings (with a variety of different characteristics) in tomographic slices are infamously common. They can have different origins related to bad (non-linear, dead) detector elements, damaged or dirty scintillator screens, and fluctuating background beam profiles. These possible different causes all impair an accurate flat-field correction leading to sinograms contaminated by vertical lines, back-projecting to circles in tomographic reconstructions. Both implemented routines for the mitigation of these artifacts work in the sinogram domain. The first approach, based on [20], takes advantage of the unsharp mask filter idea. The second technique [21] decomposes the sinogram in the wavelet/FFT domain so as to clearly separate the artifacts from real features. In this way, the artifact contribution is collapsed along the abscissa in the Fourier space where it can be easily suppressed. For user comfort, the pipeline offers also the possibility to just reconstruct a region-of-interest, save the results in different image formats, and reconstruct a rotated version of the scanned object. The signal-to-noise ratio and sharpness in the tomographic volume can be simply controlled by selecting different reconstruction filters (Ram-Lak, Hanning, Parzen,…) and adjusting their cut-off frequency.

#### Phase contrast

##### Propagation-based phase contrast

Single distance propagation-based phase contrast, a technique exploiting the coherence of synchrotron radiation, is highly utilized by the user community at TOMCAT. Its experimental simplicity (no specific hardware required) coupled to computationally efficient phase retrieval algorithms and significant contrast-to-noise (and dose) ratio improvement in tomographic volumes [22], makes it a very appealing tool and about 50% of the TOMCAT users take advantage of it. Phase contrast imaging is particularly suited to investigate biological samples characterized by small cross sections for hard X-rays. It is also a very powerful method for increasing contrast in samples composed of materials with a similar X-ray linear attenuation coefficient and is increasingly exploited also for material science applications. It has also been shown that phase retrieval (requiring projections at one single distance) can largely compensate for sub-optimal experimental conditions, such as low photon counts typical for time-resolved experiment [22] and is a fundamental tool for the study of dynamic processes.

The modular design and implementation of the pipeline facilitates a posteriori integration of different phase retrieval algorithms as simple Python functions. Currently available are routines based on the Paganin [23] (with a deconvolution step partially restoring the deteriorated spatial resolution [24]), the MBA [25], and the Moosmann [26] approach.

##### Grating interferometry

In contrast to simple single distance phase retrieval techniques, grating interferometry provides quantitative information on the electron density distribution in a sample with a higher sensitivity [27], albeit requiring a dedicated rather complex setup and still calling for multiple projections at each angular position. These multiple projections encode information not only on the electron density distribution but also on the absorption and scattering properties of the investigated specimen. This complementary information can be separated by a pixelwise FFT analysis.

Such an X-ray grating interferometer is installed at the TOMCAT beamline [28] and the required data manipulations and calculations prior to tomographic reconstruction are integrated in the pipeline. For grating interferometry data, the post-processing pipeline includes an additional step before the sinogram generation, delivering 3 sets of tomographic projections based on 3 complementary contrast mechanisms: absorption, differential phase (DPC), and dark field. This stage is parallelized by distributing the computation for each angular position to individual cores. A wavelet-FFT filter [21] is used to remove residual horizontal stripes (related to beam vibrations) from the DPC projections to guarantee highest reconstruction quality. These 3 datasets are then independently reconstructed following the traditional steps described above, using dedicated filters (e.g., Hilbert filter for DPC reconstruction), if necessary. The entire process can be launched with one single command, where the contrast of interest can be specified.

### Software technologies

Most of the pipeline code is written in Python, compatible with both the Enthought [29] and Anaconda [30] distribution. Python might not provide the ultimate computational speed and has some drawbacks (e.g., Global Interpreter Lock) in comparison for example to C. It is however very flexible, intuitive, and does not require compilation, which are the characteristics that will promote the further development of the code to integrate new routines necessary to address new problems and needs, even by non-expert programmers such as beamline staff, after the initial implementation phase. Python provides a large selection of fast, reliable, and easy-to-use scientific libraries. The pipeline implementation was for instance facilitated using the *PyWavelets* [31] and the more general *NumPy* libraries. The *NumPy* array broadcasting technology is extensively used for standard arithmetic operations guaranteeing C-like performance.

Raw data in TIFF or preferably for highest performance in HDF5 format are read using the tifffile [32] and *h5py* [16] libraries, respectively.

Parallelization at the different stages of the pipeline is achieved using the Python implementation of the message passing interface (MPI for Python (*Mpi4Py*) [33]). The pipeline software can be run on a multi-core single machine and also take advantage of high performance computing facilities. To have access to such facilities and also to optimally exploit the available computational resources on dedicated clusters, a batch-queuing system is mandatory. Our implementation works with both sun grid engine (SGE—being discontinued) and SLURM (simple linux utility for resource management [34]). These cluster management and job scheduling systems are responsible for accepting, scheduling, dispatching, and managing the distributed execution of a large number of different jobs, including job arrays. Job dependencies can be defined too. They also manage and schedule the allocation of distributed resources such as processors, memory, and disk space. Different priorities for different jobs can be defined: on a dedicated beamline cluster with simultaneous multiple users, it is possible to take advantage of the computational resources for offline calculations, without significantly affecting the performance of jobs related to an ongoing experiment.

### Hardware

The TOMCAT beamline runs few dedicated small clusters with a total of more than 100 cores, with different queues and priorities. At the Paul Scherrer Institute, 2 additional larger scale computational facilities (more than 700 cores) can also be accessed via a queue system. The newer one will be opened (also remotely) to the user community. The post-processing pipeline can be deployed on all these different systems, in an almost transparent way for the standard user.

The nodes of each cluster are interconnected by InfiniBand. To optimally exploit its power, the size of the dispatched MPI packages should be at least of few MB. InfiniBand is also used for connecting the nodes to the gpfs storage, making the time spent for I/O operations negligible compared to the overall run time.

### Graphical user interface (GUI)

**Fig. 3:**
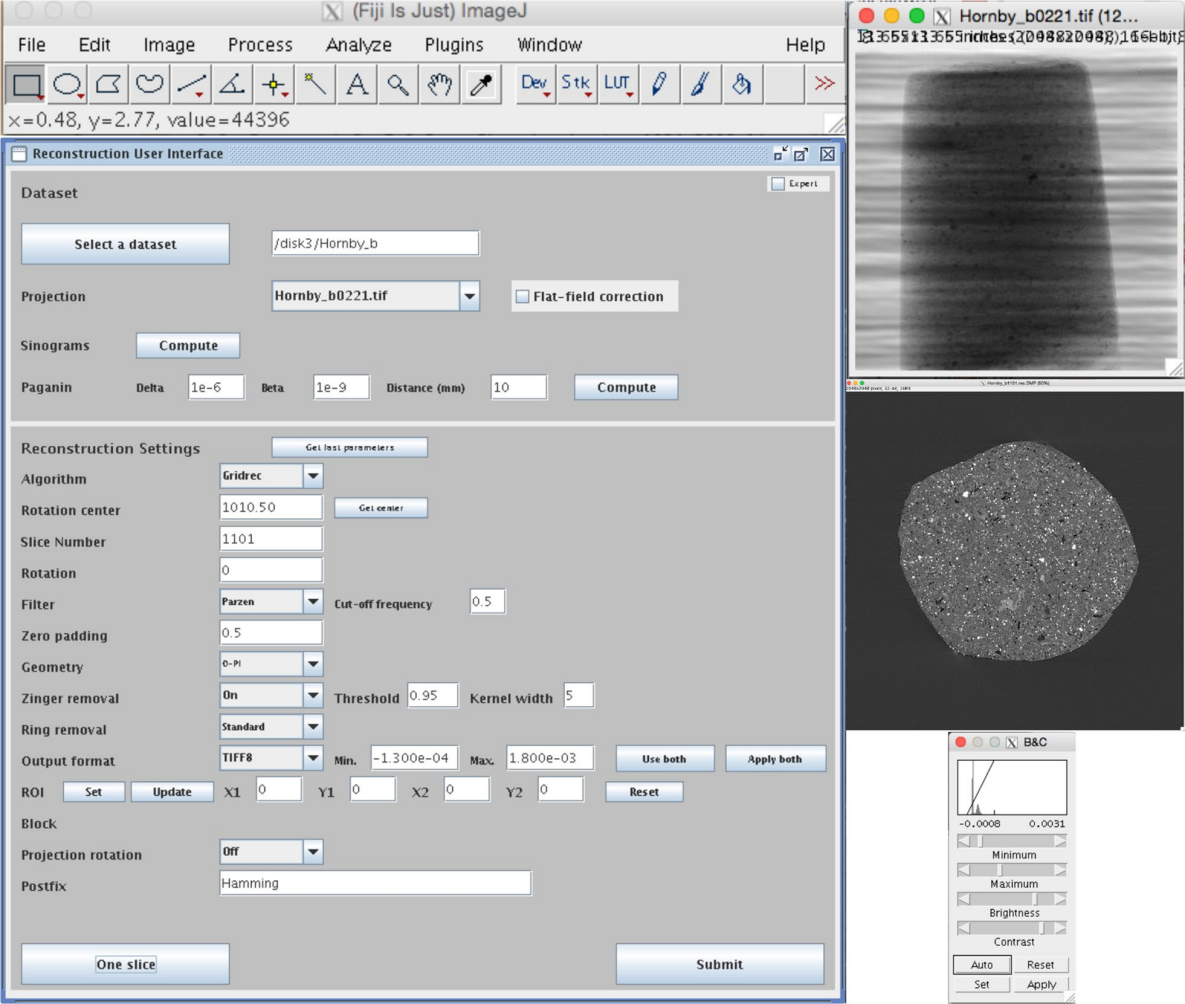
Graphical user interface (*large panel* on the *left*) enabling parameter optimization and job submission to a cluster facility for the reconstruction of full 3D volumes without the need for complex and error-prone command line activity. It is implemented as a Fiji plugin (Fiji main menu *top left*): all Fiji tools (e.g., contrast optimization tool *bottom right*) are available for projection (*top right*) and reconstruction (*middle right*) quality evaluation

The microtomography user community is very broad and the beamline users have a very diverse IT knowledge and experience, going from standard Windows users (most common) familiar with menus and buttons to computer experts (rare). To facilitate the independent reconstruction of the tomographic data by the users, without continuous support from the beamline staff, we have developed a simple graphical user interface (GUI) (Fig. 3). It enables easy tweaking of phase retrieval and reconstruction parameters and submission of the full reconstruction of a standard tomographic dataset to the computing cluster, without the need for any command line commands, usually prone to error. The users do not need to know and understand where and in which format the raw data are stored. They also do not have to be familiar with high performance computing: clicks on few buttons are enough for reconstruction optimization and submission. For more complex dynamic experiments, for instance those that produce single HDF5 files with multiple datasets, the current GUI is not adequate and reconstruction via command line is still necessary. Work is ongoing to standardize the scripts steering ultrafast experiments and the data acquisition in these more elaborated cases. This standardization should help the extension of the current GUI for the most common time-resolved experiments.

The GUI is written in Python/Jython and has been developed as a plugin for Fiji [35]. It has been necessary to implement only the aspects strictly related to the post-processing pipeline, while common tools for image analysis (histogram plot, line profile, filters, contrast enhancement,…) are readily available from the Fiji package.

## Results and discussion

### Performance

#### General considerations

To assess different performance aspects of the reconstruction pipeline, a selection of 4 real datasets, covering different experimental typologies routinely performed at the beamline, has been used (Table 1). The first 2 datasets (Ultrafast and Fast) are proxies for dynamic studies. The total acquisition time for Ultrafast was less than 50 ms, for Fast just few seconds. The other 2 datasets stand instead for standard tomographic experiments with medium (Standard) and large size (Highres) sensors. In this case, the typical total acquisition time is of 5–10 min.

**Table 1:** Dataset characteristics

Dataset name	Image size (pixels)	Number of projections	Data format	Acquisition time
Ultrafast	816 × 616	461	TIFF/HDF5	<50 ms
Fast	2016 × 1008	910	HDF5	Few s
Standard	2048 × 2048	1441	TIFF/HDF5	5–10 min
Highres	2560 × 2160	1801	TIFF	5–10 min

A dedicated cluster with 4 nodes has been used for the performance assessment. Each node has 2 Intel Xeon processors clocked at 2.70 GHz, with 256 GB RAM and 12 cores.

Table 2 presents the time required for the tomographic reconstruction of the different datasets listed in Table 1. The measured wall-clock time includes reading from and writing the data to storage, while not considered are MPI initializations and the import of the different Python modules. The total reconstruction time is split into the time required for the sinogram generation and the reconstruction part itself. When possible, the reconstruction has been performed starting from projections in TIFF and HDF5 format. For standard tomography datasets, on the used medium size cluster, the reconstruction job lasts about 1 min or less and is significantly faster than the acquisition part. A fully reconstructed dataset can therefore be visualized shortly after the end of a scan enabling quick beamline and experimental parameter assessment as well as image quality confirmation. During a beamtime, the acquisition and reconstruction process can easily proceed in parallel ensuring that at the end of an experiment all data are ready to be delivered to the users, without the need for longer stays at the facility. For dynamic experiments, the reconstruction process is currently an order of magnitude slower than the acquisition. Full 3D volumes can however be previewed few seconds after a scan guaranteeing fast feedback for instance about the beamline and experimental settings. Since dynamic studies are usually experimentally quite complex (e.g., in-situ devices) with adjustments to the setup often required, the actual acquisition time is significantly smaller than the available beamtime. Also for these experiments with bursts of high data rates leading to tens of TB of data, the post-processing pipeline ensures fully reconstructed volumes at the end of 2–3 days of beamtime.

**Table 2:** Reconstruction time of different tomographic volumes

Dataset	TIFF (s)			HDF5 (s)		
	Sinogram	Reconstruction	Total	Sinogram	Reconstruction	Total
Ultrafast	3.8	1.0	4.8	2.7	1.0	3.7
Fast	–	–	–	6.6	6.0	12.6
Standard	17.7	14.7	32.4	10.2	15.2	25.4
Highres	26.5	50.2	76.7	–	–	–

As expected the time required for the pure reconstruction job is independent from the projection format. The speed of the sinogram generation can instead strongly profit from an optimized format choice. If the projections are stored in one single HDF5 file, the sinogram generation can be sped up by about 50% compared to the case where the projections are individually stored in TIFF files. This significant improvement takes advantage of the optimization of modern shared file systems for access to large files and large chunks (MBs) of data.

Phase retrieval requiring projections at one single distance (e.g., [23]) is an invaluable tool to improve the contrast-to-noise ratio [22] often required for the segmentation and quantitative analysis of data acquired during time-resolved experiments. Table 3 summarizes the time required for phase retrieval for the datasets listed in Table 1. Projections in dynamic experiments (Ultrafast and Fast) are typically smaller and fewer than in standard high resolution experiments (Standard and Highres). Phase retrieval for the former case requires only a fraction of the total reconstruction time. For standard tomographic datasets, the phase retrieval time becomes larger than the reconstruction time, although it is less than 2 min even for the large sensor case (Highres). The total reconstruction time, also when phase retrieval is needed, remains smaller than the typical acquisition time of standard and high resolution datasets ensuring prompt post-processing of the acquired data during beamtime.

**Table 3:** Time needed for phase retrieval [23] for different datasets

Dataset	Phase retrieval time (s)	
	Single projection	Full dataset
Ultrafast	0.3	1.4
Fast	0.6	6.6
Standard	6	85.5
Highres	6	106.6

The phase retrieval algorithm works independently on single projections. The total required time scales therefore linearly with the number of projections and is inversely proportional to the number of available cores. The time for the phase retrieval of one single projection is dominated by the required 2D FFT whose complexity is O (*N* log(*N*)), with *N* the number of pixels in one projection. Projections are always padded to the nearest higher power of 2 image size to comply with the requirements of typical FFT routines, guaranteeing highest computational performance. This padding explains the equal time required for the phase retrieval of single projections for the Standard and Highres datasets.

#### Scaling properties

The post-processing pipeline can be easily deployed on different systems, from a single node machine to high performance computing clusters with hundreds of cores. To design an optimized strategy for the reconstruction of multiple datasets exploiting at best the available resources, the scaling properties of the post-processing pipeline have been analyzed in detail.

**Fig. 4:**
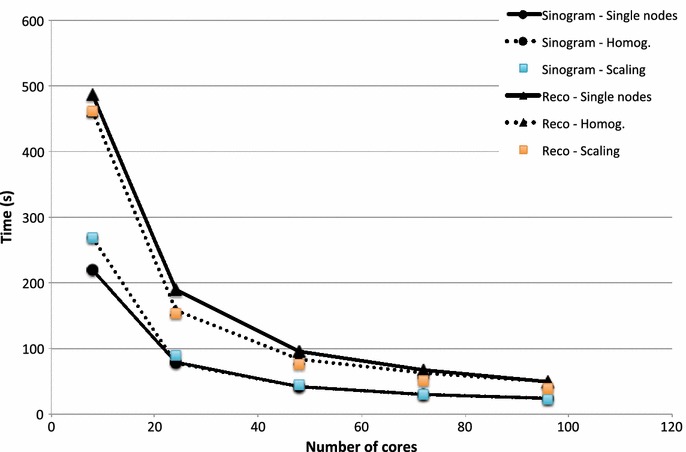
Pipeline scaling properties: time (in s) required for the sinogram generation (*circle*) and tomographic reconstruction (*triangle*) as a function of the number of used cores for 2 different computational resource configurations [used cores homogenously distributed on all available nodes (*dashed line*) and used cores concentrated on as few nodes as possible (*solid line*)]. The *square symbols* illustrate the behavior of a perfect scaling system (*blue* and *orange* for the sinogram generation and the tomographic reconstruction, respectively)

Figure 4 shows the time required for the sinogram generation and the actual reconstruction for the Highres dataset as a function of the number of cores used. Two different configurations have been used. In one case, the number of used cores is homogeneously distributed among all nodes, leaving some cores idle on each node when the full resources have not been requested. In the other case, the used cores are chosen on as few nodes as possible. With this configuration, the cores on the used nodes are all busy (except for one node if the requested cores are not a multiple of the cores per node), while the cores on the unused nodes are all idle. The results show that for the sinogram generation the difference between these two configurations is marginal except when few cores are requested, where the utilization of 8 cores on one single node would be more favorable than distributing the jobs among 4 available nodes. For the reconstruction part, homogeneous distribution of the load is instead always advantageous with performance improvements up to 20%. Considering the pipeline parallel architecture, clustering all requested cores on as few nodes as possible is expected to perform better, since in this case it is not necessary to move data to a different memory address once they have been read in. The reading and computing cores are all on the same node. This is however not what is observed in practice, where more aspects than just the parallel architecture have to be taken into account. Modern shared parallel file systems as gpfs work most efficiently if the load is shared between many different nodes. The net result favors a homogeneous distribution of the cores among the available nodes.

Figure 4 also shows that the post-processing pipeline scales well with the number of cores, with the sinogram generation almost perfectly matching the theoretical expectations. The performance of the reconstruction step is instead slightly sub-optimal, with a deteriorating yield (in the order of 25%) with the increasing number of cores, to be ascribed to the saving step of the reconstructed slices to file.

Depending on whether peak or average computational performance is more important, the available computational resources can be configured in different ways. If one single dataset has to be reconstructed in the fastest way possible, all available cores should be assigned to this one job. If the reconstruction speed of a series of hundreds of datasets, as typical for dynamic experiments, needs instead to be optimized, requesting just few nodes for the reconstruction of each volume and post-processing several datasets simultaneously is also a viable alternative solution.

#### Direct data streaming

For optimal performance of the entire acquisition–reconstruction workflow, the post-processing pipeline can also accept a ZMQ [36] stream directly from a detector instead of reading data from file. This alternative non-file-centric approach ensures a performance not influenced by the capabilities of the used shared file system and complete independence from the restrictions of the different file formats. An HDF5 file can for instance only be read once it has been closed, i.e., once the measurement has been terminated, unless the new SWMR (single-writer multiple-reader) feature is used. Direct streaming enables instead the transfer of the data in memory during the measurement and immediate start of pre-processing steps (e.g., dark and flat fields averaging) once the relevant data are available. Since our implementation is based on the PUB/SUB messaging pattern, the data can be distributed to an arbitrary number of subscribers. In our case, the raw data can therefore be simultaneously streamed to the pipeline and written to disk.

Although this feature is not yet used routinely and needs further characterization and optimization, we have successfully reconstructed a 2016 × 900 pixels dataset with 1000 projections streamed with a 1 kHz rate without the need for intermediate storage on disk.

Taking advantage of this possibility, we are also developing a preview mode based on selected reconstructed slices instead of projections as typically done. Although at least at the beginning, this advanced preview will be slower than a traditional one, reconstructed slices will provide more insightful information on the ongoing dynamic experiment than projections. The post-processing pipeline can currently already deliver 20 tomographic slices for the Standard datasets only 1.4 s after receiving the last image from the ZMQ stream. For smaller datasets (e.g., Ultrafast), 13 slices are available in 0.4 s. This capability will lead to unprecedented control ability enabling more objective real-time tuning of the experimental parameters in in-situ experiments in response to the dynamic evolution of the study system, usually poorly known in advance.

## Conclusions

Sub-second tomographic microscopy at third-generation synchrotron sources is a reality and sustained elevated data rates of multiple GB/s in tomographic experiments will become even more common at diffraction-limited storage rings. The computational tools necessary for the post-processing of raw tomographic projections have generally not experienced the same efficiency increase, often leading to a strong mismatch between the speed of a tomographic experiment and the time required for the reconstruction of a 3D volume needed to assess the validity of the experiment. We present here an efficient post-processing pipeline overcoming this mismatch and delivering reconstructed tomographic datasets just few seconds after the data have been acquired, despite being optimized for a CPU architecture. It is flexible and able to handle raw data exploiting different contrast mechanisms (standard absorption contrast, propagation-based phase contrast, differential phase contrast, and dark field). This new pipeline is based on a modular framework and can easily be extended with new features even by non-expert programmers thanks to its implementation in Python. It is supplemented with a user-friendly graphical interface easing the tomographic reconstruction work mostly running in parallel with the actual experiment. The pipeline software can be deployed in a transparent way on single- and multi-node systems as well as high performance computing facilities.

In addition to reading raw data from file, the post-processing pipeline can also accept a ZMQ stream, for instance directly from the detector. This feature makes the pipeline independent from the performance of the shared file system and the intrinsic characteristics of the adopted file format. It also opens up new possibilities for objective and active control of the performed dynamic experiments, when previewing tools based on 2D tomographic slices instead of raw projections are used. Although further mathematical and computational optimization is needed to achieve a true real-time tomographic preview offering also for instance on-the-fly 3D visualization (and eventually data quantification), the presented post-processing pipeline can already provide selected tomographic slices in less than 0.4 s for typical ultrafast experiments.

The bottleneck in the entire workflow has now moved to the transfer of tens of TBs of raw and reconstructed data to the host institutions of our users, either using external hard drives, network-attached storage (NAS) devices, or per remote transfer. At the Paul Scherrer Institute on-site long-term storage possibilities are becoming available as well as remote access to a large computing facility for data quantification, another weak point in the general tomographic workflow at most facilities, starting to be addressed by the scientific community.
